# Imaging Bioluminescent Exogenous Stem Cells in the Intact Guinea Pig Cochlea

**DOI:** 10.1002/ar.24068

**Published:** 2019-02-06

**Authors:** Timo Schomann, Laura Mezzanotte, John C. M. J. de Groot, Clemens W. G. M. Löwik, Johan H. M. Frijns, Margriet A. Huisman

**Affiliations:** ^1^ Auditory Neurobiology Laboratory, Department of Otorhinolaryngology and Head and Neck Surgery Leiden University Medical Center Leiden The Netherlands; ^2^ Optical Molecular Imaging, Department of Radiology Erasmus Medical Center Rotterdam Rotterdam The Netherlands

**Keywords:** bioluminescence imaging, firefly luciferase, hair‐follicle‐bulge‐derived stem cells, cochlea, guinea pig

## Abstract

Stem‐cell‐based therapy may be used to replace damaged or lost neurons in the cochlear nerve of patients suffering from severe‐to‐profound sensorineural hearing loss. In order to achieve functional recovery in future clinical trials, knowledge about survival of grafted cells and their differentiation into functional neurons is a prerequisite. This calls for non‐invasive *in vivo* visualization of cells and long‐term monitoring of their survival and fate after cochlear transplantation. We have investigated if molecular optical imaging enables visualization of exogenous cells in the intact cochlea of guinea pig cadaver heads. Transduced (stem) cells, stably co‐expressing fluorescent (copGFP) and bioluminescent (Luc2) reporter molecules, were injected into the internal auditory meatus or directly into the cochlea through the round window. After injection of the cells into the internal auditory meatus, a bright bioluminescent signal was observed in the cavum conchae of the auricle, indicating that light generated by Luc2 is passing through the tympanic membrane and the external auditory meatus. Similar results were obtained after injection of the cells through the round window membrane, either directly into the scala tympani or in Rosenthal's canal within the modiolus of the basal cochlear turn. Imaging of the auditory bulla demonstrated that the bioluminescent signal passes through the tympanic membrane and crevices in the bony wall of the bulla. After opening the auditory bulla, the bioluminescent signal was emanating from the round window. This is the first study demonstrating that bioluminescence imaging enables visualization of luciferase‐expressing cells injected into the intact guinea pig cochlea. Anat Rec, 303:427–440, 2020. © 2019 The Authors. *The Anatomical Record* published by Wiley Periodicals, Inc. on behalf of American Association of Anatomists.

The inner ear in Mammals contains the sense organs for hearing (cochlea) and balance (vestibular system). Within the cochlea, the sensory epithelium, or organ of Corti, houses two different types of sensory cells: the outer and inner hair cells. The former function as acoustical pre‐amplifiers, whereas the latter are the actual sound receptor cells. Inner hair cells are responsible for the mechano‐electric transduction process in the cochlea; they transduce sound‐induced vibrations into a chemical signal leading to an electric signal which is delivered by the auditory nerve to the brain, where auditory information is finally processed.

Degeneration of the hair cells and spiral ganglion neurons (SGNs) in the cochlea leads ultimately to sensorineural hearing loss (SNHL). The World Health Organization has estimated that approximately 5% of the world's population suffer from some form of disabling SNHL (WHO, 2017). In humans and other mammalian species, the cochlea itself is not able to spontaneously replace lost hair cells and SGNs.

SNHL currently cannot be treated by means of either pharmaceutical or surgical intervention, but auditory function can be partially restored with conventional hearing aids or—in case of severe‐to‐profound SNHL—with a cochlear implant, which electrically stimulates the residual SGNs in the modiolus directly. Since the first application of this kind of cochlear prosthesis, technology has progressed significantly and this has led to advanced electrode and speech processor strategies resulting in higher performance levels (Bierer et al., 2011; Kalkman et al., 2015). There nevertheless exists considerable inter‐individual variability in performance, especially in noisy listening environments. This variability can be explained by regional variations in the distance between the sites of action potential initiation and the electrodes of the cochlear implant resulting from regression of the dendrites of the SGNs (Bierer et al., 2011; Kalkman et al., 2015). Alternatively, it may be due to neurocellular changes creating variation in the sensitivity of SGNs (Briaire and Frijns, 2006).

It is now generally accepted that the preservation of functional SGNs in sufficiently high numbers along the entire cochlear spiral is crucial with regard to cochlear implant performance (Gantz et al., 1993; Eppsteiner et al., 2012; Snel‐Bongers et al., 2013). Besides local loss of SGN dendrites after prolonged deafness and neural degeneration, variation in electrode position within the cochlea and/or tissue growth (fibrosis and ossification) can also reduce the performance of the cochlear implant (Briaire and Frijns, 2006; Bierer et al., 2011). In adults, not much is known about the precise histological status in the cochlea and the time course of SGN degeneration in deafness and reports are contradictory (*cf*., Snel‐Bongers et al., 2013). Even less is known about the neural status in deaf children, and probably much depends on the etiology causing the deafness. A growing body of evidence from animal studies suggests that cell‐based therapy using stem cells and/or neural progenitor cells may be applied to replace damaged or lost SGNs, and this may eventually result in enhanced cochlear implant performance in patients (Rask‐Andersen et al., 2005; Regala et al., 2005; Coleman et al., 2006; Corrales et al., 2006; Hu and Ulfendahl, 2006; Sekiya et al., 2007; Chen et al., 2012; Gunewardene et al., 2012; Huisman and Rivolta, 2012; Needham et al., 2013; Shi and Edge, 2013). However, survival of grafted stem cells or neural progenitor cells and their differentiation into SGNs and glial (Schwann) cells as well as their functional integration into extant peripheral auditory structures are a prerequisite to achieve functional repair of the cochlear nerve. This calls for non‐invasive *in vivo* visualization of grafted stem cells and longitudinal monitoring of their survival and fate in the cochleas of deafened animals.

Molecular optical imaging based on reporter gene expression is a highly sensitive and versatile imaging modality and is gaining popularity in small animal research, because it allows for real‐time tracking of different kinds of grafted cells as well as *in vivo* monitoring of the migration, proliferation and persistence of exogenous cells within the host (for reviews, see De Almeida et al., 2011; Welsh and Noguchi, 2012; Mezzanotte et al., 2017). In order to track grafted cells by means of whole‐body molecular optical imaging, it is essential that these cells stably express reporter molecules that can be visualized. Genetic modification of cells using a lentiviral construct carrying a foreign gene that codes for a fluorescent, or bioluminescent, reporter molecule is a usual approach and results in stable expression of the reporter molecule, which can then be detected by means of either fluorescence or bioluminescence imaging (Fig. 1). Fluorescence imaging is based upon the phenomenon that a fluorophore absorbs energy from a light source and emits light at a different wavelength (Shagin et al., 2004; Mezzanotte et al., 2013). Bioluminescence imaging, in contrast, is based upon the emission of light generated during the enzymatic conversion of D‐luciferin into oxyluciferin by luciferase enzymes.

**Figure 1:**
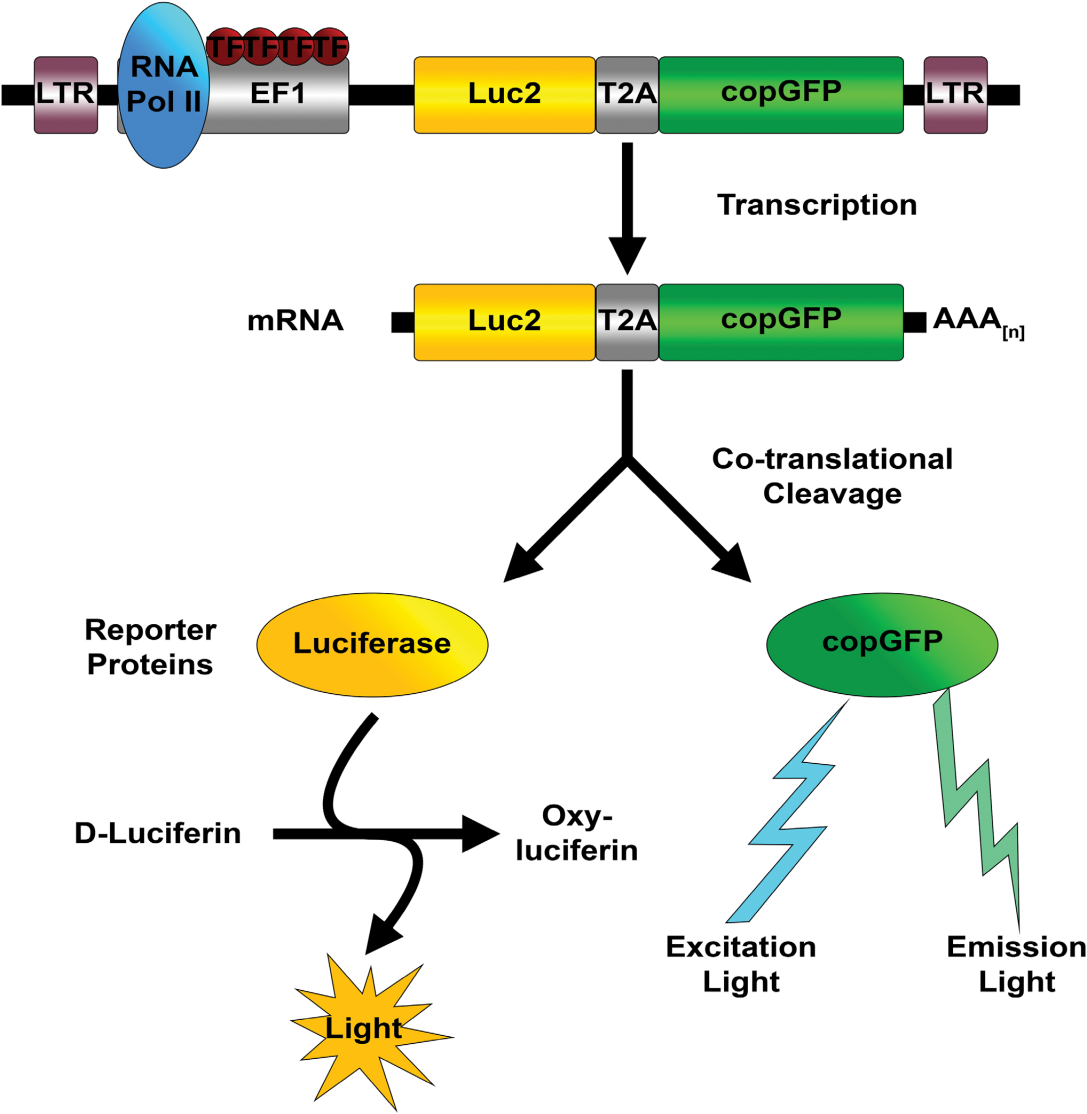
Schematic drawing explaining the basic principles of dual‐reporter gene expression in genetically engineered cells. The lentiviral gene construct is designed to stably co‐express copepod green fluorescent protein (copGFP; emitting at ∼502 nm) and codon‐optimized firefly luciferase Luc2 (emitting at ∼560 nm). The copGFP‐Luc2 construct is composed of the EF1 promotor and genes coding for copGFP and Luc2. Both genes are coupled *via* a T2A‐like sequence, which mediates co‐translational cleavage (ribosome skipping) and, hence, results in bicistronic expression. The inserts are flanked by long terminal repeats (LTR). TF: transcription factors; RNA Pol II: RNA polymerase II.

We have designed a lentiviral gene construct resulting in stable, equimolar co‐expression of a fluorescent (copGFP) and bioluminescent reporter molecule (Luc2), because such a dual‐reporter approach exploits the different but complementary advantages of both reporter molecules. Whereas the fluorescent reporter is advantageous for *in vitro* light‐microscopical detection of transduced cells and post‐mortem visualization of grafted cells in histological sections of the cochlea, the bioluminescent reporter is more suitable for detection of grafted cells using optical whole‐body molecular optical imaging, because of its high sensitivity, a high signal‐to‐noise ratio—due to low background luminescence levels—and the higher penetration depth, as compared to fluorescence imaging (Choy et al., 2003; Massoud and Gambhir, 2003; Shah and Weissleder, 2005; Zhao et al., 2010). Furthermore, as enzymatic conversion of D‐luciferin into oxyluciferin is dependent on ATP and O_2_, the bioluminescent signal can be used as a proxy for cell viability and, hence, to confirm the viability of the injected cells.

The objective of this study was to investigate if visualization of exogenous stem cells within the intact cochlea of cadaver guinea pig, using molecular optical imaging, is feasible for future *in vivo* stem cell transplantation experiments. We have used the guinea pig because it is the most commonly used animal model of deafness. However, molecular imaging in the guinea pig is particularly challenging since this species is a newcomer to the field of molecular optical imaging and because the cochlea is embedded within the auditory bulla consisting of compact bone with a high mineral density, which may block signal detection during molecular optical imaging.

## MATERIALS AND METHODS

### Animals

In order to avoid the unnecessary use of living animals, and in compliance with the 3Rs guiding principles for animal experimentation, we have only used cadavers from animals used in non‐related experiments.

Cadaver heads (*n* = 26; i.e., 52 cochleas) from adult female guinea pigs (strain: Dunkin Hartley) were obtained from the Department of Pharmaceutical Sciences (Utrecht University, the Netherlands). Animals had been used for non‐related experiments. Approval for their use was obtained from the respective Animal Welfare Officers of Utrecht University and Leiden University Medical Center (LUMC).

Surplus C57BL/6 mice served as a source for whisker pads and were obtained from the LUMC Central Animal Facility. The use of surplus mice was approved by the LUMC Animal Experiments Committee (DEC permit 10172).

### Cell Cultures

Two different types of cells were used in this study: (1) HEK293‐copGFP‐CBG99 cells, a recently established cell line that stably expresses copGFP and the CBG99 luciferase at an equimolar ratio (Mezzanotte et al., 2013); and (2) hair‐follicle‐bulge‐derived stem cells (HFBSCs), which have been proved to be multipotent and may differentiate into neurons and glial cells, both *in vitro* and in living animals (Liu et al., 2011; Gho et al., 2016). HEK293‐copGFP‐CBG99 cells were used in a series of experiments to determine the implantation approach of first choice (*cf*., section “Whole‐head imaging after cochlear implantation of transduced cells”), because these cells are routinely cultured and abundantly available at our laboratories, in contrast to the HFBSCs. We used HFBSCs, because we aim to use these stem cells in future transplantation experiments.

HEK293‐copGFP‐CBG99 cells were cultured in Dulbecco's modified Eagle's medium (DMEM; Sigma‐Aldrich, St. Louis, MO, USA) supplemented with 10% fetal bovine serum (FBS), 2 mM L‐glutamine and penicillin/streptomycin solution (diluted 1:100; PAA Laboratories, Pasching, Austria). The cell cultures were maintained in a humidified incubator with 5% CO_2_ at a temperature of 37°C.

HFBSCs were isolated from mouse whiskers according to a previously described protocol (Schomann et al., 2016). Hair follicles were dissected out from the whisker pads and the bulge region was removed. Explants were transferred to 12‐well cell culture plates pre‐coated with poly‐d‐lysine (PDL; Sima‐Aldrich, St. Louis, MO, USA), stem cell culture medium was added and stem cells were allowed to migrate from the explant. Stem cell culture medium, modified from Nguyen et al. (2013), consisted of DMEM/F12 (Biochrom AG, Berlin, Germany) supplemented with Gibco® GlutaMax™‐I (Life Technologies™), to which 10% FBS, Gibco® B‐27® serum‐free supplement (Life Technologies™), N2 supplement (R&D Systems, Minneapolis, MN, USA), epidermal growth factor (20 ng/mL; R&D Systems), basic fibroblast growth factor (20 ng/mL; R&D Systems) and antibiotic/antimycotic solution (diluted 1:100; Sigma‐Aldrich) were added.

After 1 week in culture, the explants were removed, culture medium was changed and cells were allowed to grow until they reached 70–80% confluency. Culture medium was changed twice weekly. Cell cultures were maintained in a humidified incubator with 5% CO_2_ at a temperature of 37°C. After several cycles of passage and expansion, cells were trypsinized (0.05% trypsin and 0.02% EDTA.4Na in PBS) for 2 min, pelleted by centrifugation at ∼280×*g* for 10 min, resuspended (cell density: 1 × 10^6^ cells/mL) in FBS containing 10% DMSO and immediately frozen, and stored at −80°C.

### Lentiviral Vector Production and Transduction of HFBSCs

We designed a lentiviral gene construct (Fig. 1) that allows stable, equimolar co‐expression of copGFP and Luc2. Details on cloning and recombination procedures have been reported previously (Mezzanotte et al., 2013). In brief, the gene coding for Luc2 is inserted into the multiple cloning site (MCS) of the lentiviral expression vector pCDH‐EF1‐MCS‐T2A‐copGFP (SBI System Biosciences, Mountain View, CA), which contains the elongation factor 1α (EF1) promoter and a T2A‐like sequence. The T2A peptide mediates co‐translational cleavage allowing bicistronic expression (Szymczak and Vignali, 2005).

Lentivirus particles were generated by means of calcium‐phosphate‐mediated transfection in HEK293 cells (Abbas et al., 2000). Virus was quantified by antigen‐capture ELISA, measuring HIV p24 levels (ZeptoMetrix Corporation, Buffalo, NY). These values were converted to an infectious titer using the approximation that 1 ng of p24 equals 2,500 infectious units. HFBSCs were resuspended in medium and subsequently transduced with pseudoviral particles containing the copGFP‐Luc2 construct, using 40 ng virus per 1 × 10^5^ cells, followed by storage at −80°C.

Lentiviral vector production and stem cell transduction were performed under appropriate biosafety level conditions (ML‐II) in accordance with the National Biosafety Guidelines and Regulations for Research on Genetically Modified Organisms. Procedures and protocols were reviewed and approved by the LUMC Biosafety Committee (GMO permit 00‐026).

### Loading of Transduced HFBSCs with Iron Oxide Nanoparticles

HFBSCs containing the copGFP‐Luc2 construct were loaded with superparamagnetic iron oxide nanoparticles (ferumoxytol) according to the procedure of Thu et al. (2012). An amount of 4 × 10^6^ cells were resuspended in serum‐free basic growth medium (BGM) containing 2 IU/mL sodium heparin (LEO Pharma, Amsterdam, the Netherlands), 60 μg/mL protamine hydrochloride (MEDA Pharma BV, Amstelveen, the Netherlands) and 50 μg/mL ferumoxytol (Takeda Pharma A/S, Roskilde, Denmark) followed by incubation at 37°C for 2 hr. An equal amount of BGM containing 20% FBS was added, upon which the cells were transferred to PDL‐coated dishes and incubated in a humidified incubator with 5% CO_2_ at a temperature of 37°C. After 24 hr, the cells were washed with PBS and then with PBS containing heparin (10 IU/mL). The cells were passaged by adding pre‐warmed (37°C) balanced salt solution containing 0.05% trypsin and 0.02% EDTA.4Na (Gibco Life Technologies) to the culture dish and incubation for 2 min. Cells were collected and resuspended in PBS at a concentration of 5 × 10^4^ cells/μL and stored at 4°C until transplantation.

### *In vitro* Light‐Microscopical Detection of Reporter Molecule Expression in Transduced HFBSCs

#### *Fluorescence microscopy*

Transduced HFBSCs containing the copGFP‐Luc2 construct were plated and allowed to attach in PDL‐coated 12‐well cell culture plates. Real‐time observation of copGFP expression was performed using an Olympus IX70 epi‐illumination fluorescence microscope (FITC filter settings) equipped with a Leica DFC340 FX digital color camera. Images were acquired and digitally stored using Leica Application Suite Advanced Fluorescence (LASAF) version 1.9 software.

#### *Bioluminescence microscopy*

HFBSCs with the copGFP‐Luc2 construct were plated and allowed to attach in PDL‐coated glass‐bottom microwell dishes (MatTek Corporation, Ashland, MA, USA) containing 3 mL of stem cell culture medium. Luc2 expression was assessed in real time using bioluminescence microscopy (Buijink et al., 2016). For this purpose, an Olympus BX51WIF microscope was fitted with a V240 XY‐shifting table (Luigs & Neumann, Ratingen, Germany) and a Hamamatsu ORCA UU‐BT‐1024G high‐resolution, back‐thinned CCD camera, and the microscope's main body was enclosed by a custom‐made dark box to block external light. D‐luciferin (potassium salt; Synchem, Felsberg, Germany) was added at a final concentration of 0.1 mM, i.e., 10‐fold lower than that used by Ogoh et al. (2014). The bioluminescent signal was recorded over a course of 15 min, during which the cells were kept at a temperature of 37°C. Images were acquired and digitally stored using Image‐Pro® Plus software, followed by conversion of the grayscale images into pseudocolor images.

#### In vitro *imaging of luciferase expression in transduced HFBSCs*

In order to determine the time window for optimal signal measurement, transduced HFBSCs containing the copGFP‐Luc2 construct were resuspended in PBS and plated in different amounts (2.5 × 10^4^/100 μL or 5 × 10^4^ cells/100 μL per well) in black‐walled 96‐well plates (NUNC™, Rochester, NY, USA). D‐luciferin was added at a final concentration of 0.5 mM and bioluminescence was recorded by consecutive 30‐sec acquisitions during 30 min. All images were taken with the IVIS® Spectrum multimodal imaging system (Xenogen, Caliper Life Sciences, Hopkinton, MA, USA) using an open filter, field of view C (default setting), f/stop = 1, and medium binning for all bioluminescence measurements. Image acquisition and analysis were done with *Living Image* version 4.2.1 software (Caliper Life Sciences, Hopkinton, MA).

#### *Whole‐head imaging after cochlear implantation of transduced cells*

Real‐time observations were performed either on intact auditory bullae after removal from the cadaver heads or on auditory bullae left *in situ* within the cadaver heads. Cells were resuspended in PBS at a density of 1 × 10^6^ cells/100 μL. After dilution and addition of D‐luciferin (at a final concentration of 15 mg/mL), 5 μL of the cell suspension was injected using a 20‐μL Hamilton syringe with a 25‐gauge needle. The volume of the perilymphatic injections was less than the total volume (8–10 μL) of perilymph in the guinea pig cochlea (Thorne et al., 1999; Shinomori et al., 2001). Cell suspensions were also injected into the internal auditory meatus (volume: 10 μL) and into the cochlear modiolus at a volume of ≤5 μL (Corrales et al., 2006; Ogita et al., 2009; Chen et al., 2012).

All imaging measurements were performed with the IVIS® Spectrum multimodal imaging system. Digital images were acquired immediately after injection of the cell suspensions into the cochlea, approximately 15 min after addition of D‐luciferin to the cell suspension. A grayscale image of the cadaver head was first collected, using the laser scan surface topography modality, followed by the acquisition and overlay of the pseudocolor bioluminescent images. Image acquisition and analysis were done with *Living Image* version 4.2.1 software using a 30‐sec acquisition time, open filter, field of view C (default setting), f/stop = 1, and medium binning for all bioluminescence measurements.

The imaging experiments were performed at the Small Animal Imaging Unit of the LUMC Central Animal Facility under appropriate biosafety level conditions (DM‐II) in accordance with the National Biosafety Guidelines and Regulations for Research on Genetically Modified Organisms.

#### *Validation of cochlear implantation of transduced cells*

To determine if the reporter signal passes through the bony capsule of the cochlea, auditory bullae (*n* = 4) were removed from the cadaver heads and HEK293‐copGFP‐CBG99 cells (5 × 10^4^ cells in 10 μL luciferin‐containing PBS) were injected directly into the internal auditory meatus followed by imaging of the intact bullae. Next, the auditory bullae were opened to reveal its internal structures and the cochleas were viewed by imaging.

In order to confirm if cells injected into the internal auditory meatus actually reach Rosenthal's canal in the cochlear modiolus, 10 μL of a 1% aqueous solution of methylene blue were injected into the internal auditory meatus. After 30 min, the cochleas (*n* = 2) were removed from the auditory bulla, fixed in 2.5% glutaraldehyde in 0.1 M sodium cacodylate/HCl buffer (pH 7.4) for 1 hr, rinsed in the same buffer and decalcified in 10% EDTA.2Na (pH 7.4) at room temperature for 1 week. Next, the decalcified cochleas were dehydrated in an ascending ethanol series and treated according to the Spalteholz clearing method using methyl salicylate and benzyl benzoate (Voie, 2002) to render the tissues and decalcified bone transparent. Specimens were examined with a Leica M205C stereomicroscope equipped with a Leica IC80 HD color camera. Digital images were acquired and stored using Leica Application Suite (LAS V4.5; Leica Camera AG, Wetzlar, Germany) software.

#### *Comparison of application routes for cochlear implantation*

**Figure 2:**
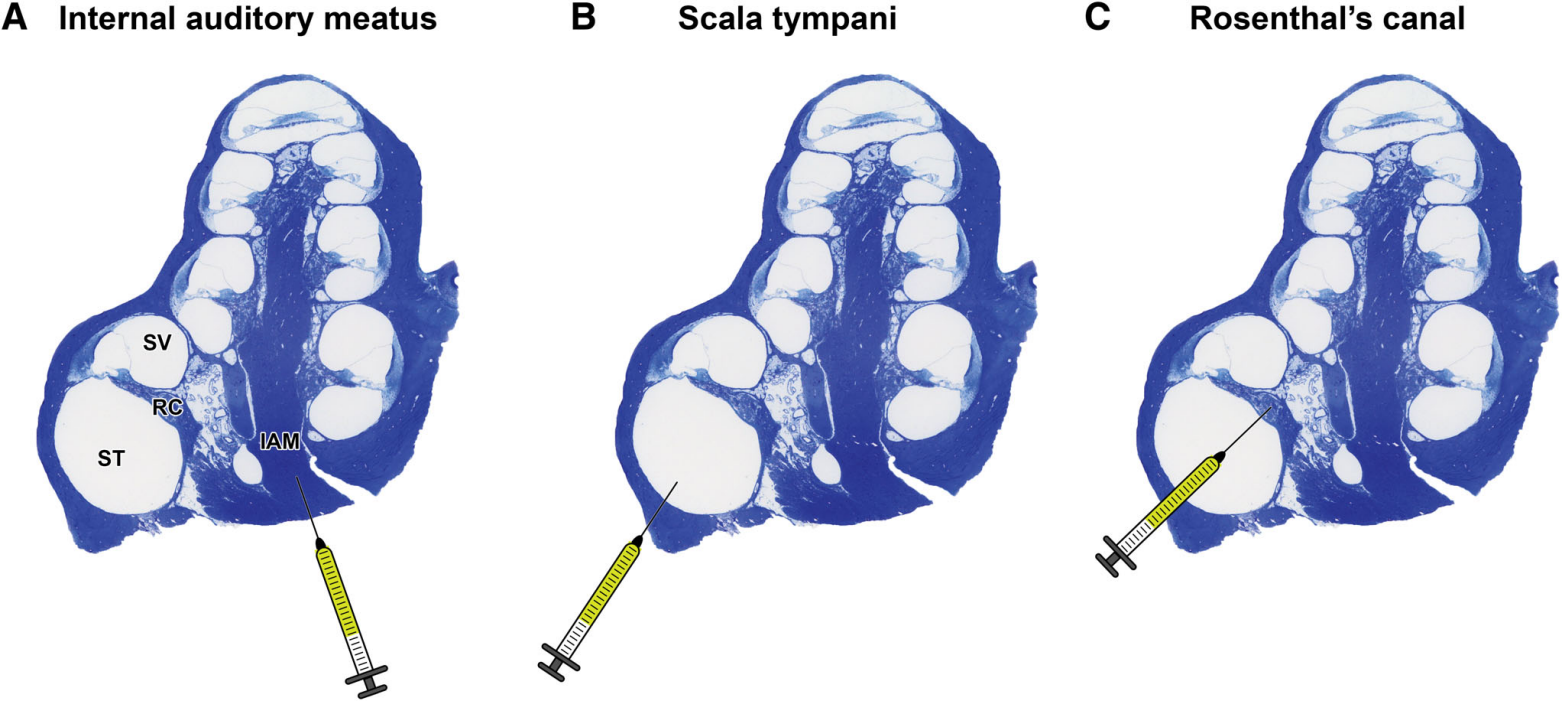
Illustration of the three different injection routes. (**A**) Injection into the internal auditory meatus. (**B**) Injection into the scala tympani *via* the round window membrane. (**C**) Injection into Rosenthal's canal *via* the round window membrane. IAM: internal auditory meatus; ST: scala tympani; SV: scala vestibuli; RC: Rosenthal's canal.

Three different application routes were compared, in order to determine the most convenient approach to introduce transduced cells into the cochlea (Fig. 2). To this end, the auditory bullae were left *in situ* in the cadaver heads.

With the first approach (Fig. 2A), the foramen magnum was opened and widened to expose the internal auditory meatus located in the medial aspect of the auditory bulla. HEK293‐copGFP‐CBG99 cells (5 × 10^4^ cells in 10 μL PBS containing D‐luciferin at a final concentration of 15 mg/mL) were injected directly into the internal auditory meatus of the right ear (*n* = 4). The left ear (*n* = 4) was not injected and served as control.

During the second approach (Fig. 2B), a retro‐auricular surgical incision was made to expose the skull bone and the right auditory bulla (*n* = 4) was opened with a diamond burr to obtain easy access to the round window niche. HEK293‐copGFP‐GCB99 cells (5 × 10^4^ cells in 5 μL luciferin‐containing PBS) were injected directly into the scala tympani through the round window membrane. The left ear (*n* = 4) served as control.

With the third approach (Fig. 2C), HEK293‐copGFP‐CBG99 cells (5 × 10^4^ cells in 5 μL luciferin‐containing PBS) were injected into Rosenthal's canal through the round window membrane of the right cochlea (*n* = 4). The left ear (*n* = 4) served as control.

As an additional control for all approaches, cells were injected into the left masseter muscle.

#### *Cell dilution series*

In addition, a cell dilution series was performed to determine the amount of cells needed to reach signal threshold for bioluminescent imaging. Transduced HFBSCs containing the copGFP‐Luc2 construct were resuspended in PBS at a density of 1 × 10^6^ cells per 100 μL. After appropriate dilution and addition of D‐luciferin (at a final concentration of 15 mg/mL), different volumes (10, 5, 2.5, and 1 μl) containing 5 × 10^4^ cells, 2.5 × 10^4^ cells, 1.25 × 10^4^ cells, and 0.5 × 10^4^ cells, respectively, were injected into Rosenthal's canal through the round window membrane (*cf*., section “Comparison of application routes for cochlear implantation”). Both the right (*n* = 8) and left cochleas (*n* = 8) were injected, but the left cochleas received the lower amount of cells.

#### *Detection of transduced iron‐containing HFBSCs in histological sections of cochleas*

To visualize HFBSCs in histological sections of the cochlea, auditory bullae (*n* = 6) were used to inject transduced HFBSCs containing iron oxide nanoparticles (5 × 10^4^ cells in 5 μL luciferin‐containing PBS) into the scala tympani through the round window membrane (*cf*., section “Comparison of application routes for cochlear implantation”).

Immediately following imaging, the cochleas were removed from the auditory bullae and fixed by intralabyrinthine perfusion with 4% formaldehyde in 0.1 M sodium cacodylate buffer (pH 7.4) and overnight immersion in the same fixative at 4 °C. Next, the cochleas were rinsed in the same buffer and decalcified in 10% EDTA.2Na (pH 7.4) at room temperature for 1 week. Decalcified cochleas were dehydrated in an ascending ethanol series and xylene followed by paraffin embedding. Serial sections were cut at a thickness of 8 μm along a plane parallel to the central axis of the modiolus, mounted on gelatin‐coated glass slides, dewaxed in xylene and rehydrated in a descending ethanol series.

Sections were pre‐treated with 3% H_2_O_2_ in methanol for 30 min to inhibit endogenous peroxidase activity and washed in distilled water (30 min). Iron‐containing nanoparticles were visualized using Perls' Prussian blue method followed by DAB intensification (Meguro et al., 2007). Incubation in 1% potassium ferrocyanide [K_4_Fe(CN)_6_.3H_2_O] in 1% aqueous hydrochloric acid for 30 min was followed by several washes in distilled water (3 × 10 min). Next, the sections were incubated in a solution containing 0.1% 3,3′‐diaminobenzidine.4HCl and 0.03% H_2_O_2_ in PBS in the dark for 10 min, followed by three washes in distilled water (5 min each) to stop the reaction. The sections were subsequently mounted in Roti®‐Mount FluorCare (Carl Roth GmbH + Co. KG, Karlsruhe, Germany) mounting medium and examined with a Leica DM5500B microscope equipped with a Leica DFC 450C color camera. Digital images were acquired and stored using Leica Application Suite (LAS V4.5; Leica Camera AG, Wetzlar, Germany) software.

## RESULTS

### Detection of Light Emanating from the Cochlea

**Figure 3:**
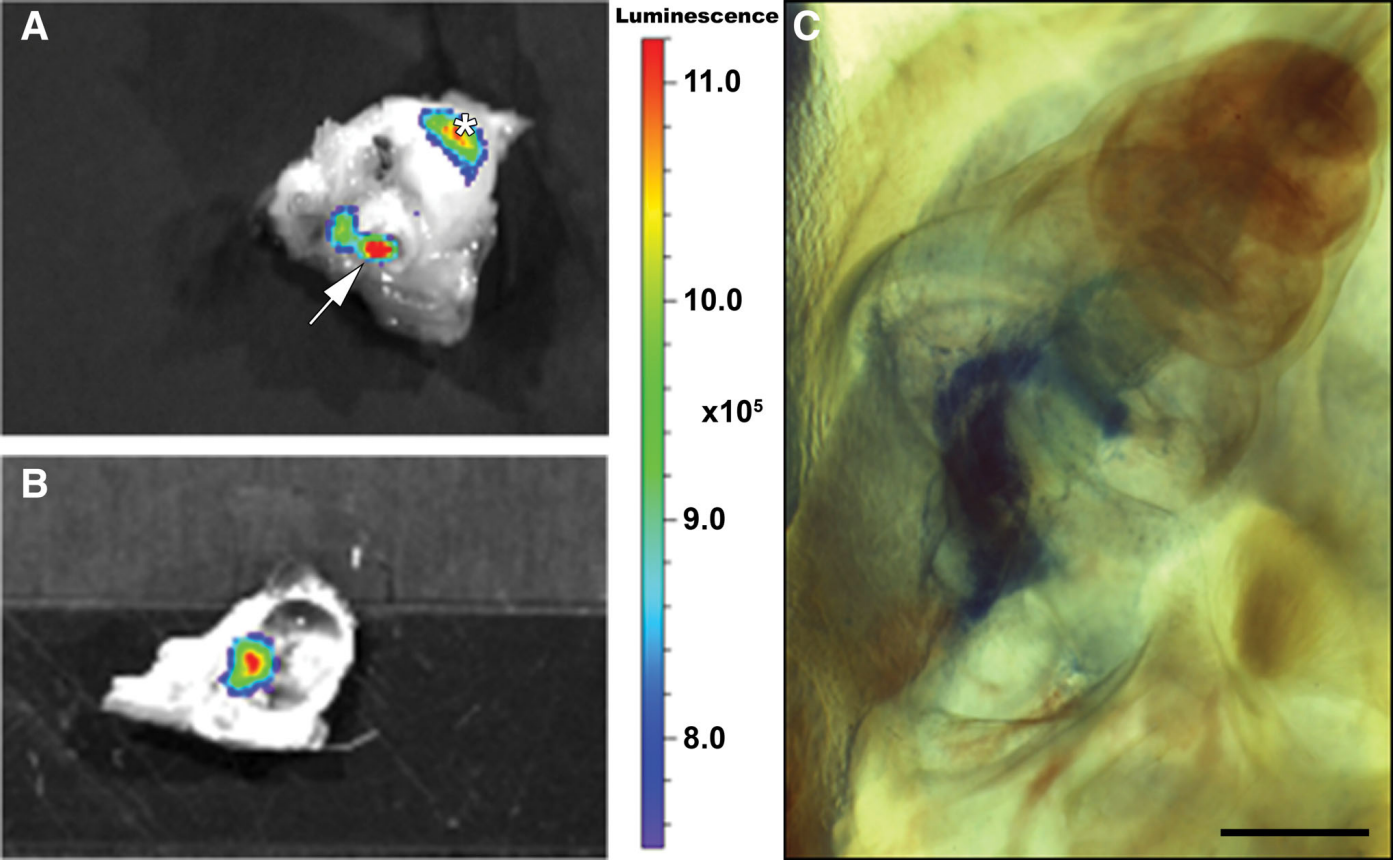
Detection of bioluminescent signals emanating from the cochlea *in situ* and verification of injection. (**A**, **B**) Pseudocolor light emission images of an auditory bulla after injection of HEK293‐copGFP‐CBG99 cells (5 × 10^4^ cells in 10 μL PBS containing D‐luciferin at a final concentration of 15 mg/mL) directly into the internal auditory meatus. Bioluminescence is expressed as radiance (photons/second/cm^2^/sr). Optical imaging of the auditory bulla after its removal from the skull demonstrates that the bioluminescent signal can pass through the tympanic membrane (arrow) and crevices (*) in the bony wall of the auditory bulla (**A**). After removing part of the bony wall of the auditory bulla (**B**), the cochlea can be discerned and a bright bioluminescent signal is observed at the basal cochlear turn, localized near the round window. (**C**) Photomicrograph of a cleared cochlea after injection of a methylene blue solution directly into the internal auditory meatus. Myelin sheaths of the neurons in the basal and middle turns are stained blue, allowing visualization of Rosenthal's canal (scale bar: 1 mm).

In order to determine if it is possible to detect bioluminescent signals emanating from the cochlea *in situ*, HEK293‐copGFP‐CBG99 cells were injected into the internal auditory meatus of intact auditory bullae. A bright and distinctly localized bioluminescent signal could be seen passing through the tympanic membrane and crevices in the bony wall of the auditory bulla (Fig. 3A). The signal was observed emanating from the round window after opening the bulla and exposing the cochlea (Fig. 3B). This observation indicates that HEK293‐copGFP‐CBG99 cells, after injection into the cochlea, retain enough luciferase activity to yield detectable bioluminescence.

In order to confirm if cells injected into the internal auditory meatus actually reach Rosenthal's canal in the modiolus, we injected cochleas with methylene blue. In cleared specimens a distinct blue staining of the myelin sheath of the auditory neurons was present in the basal and middle turns (Fig. 3C).

### Comparison of the Different Application Routes

#### *Approach* via *the internal auditory meatus*

**Figure 4:**
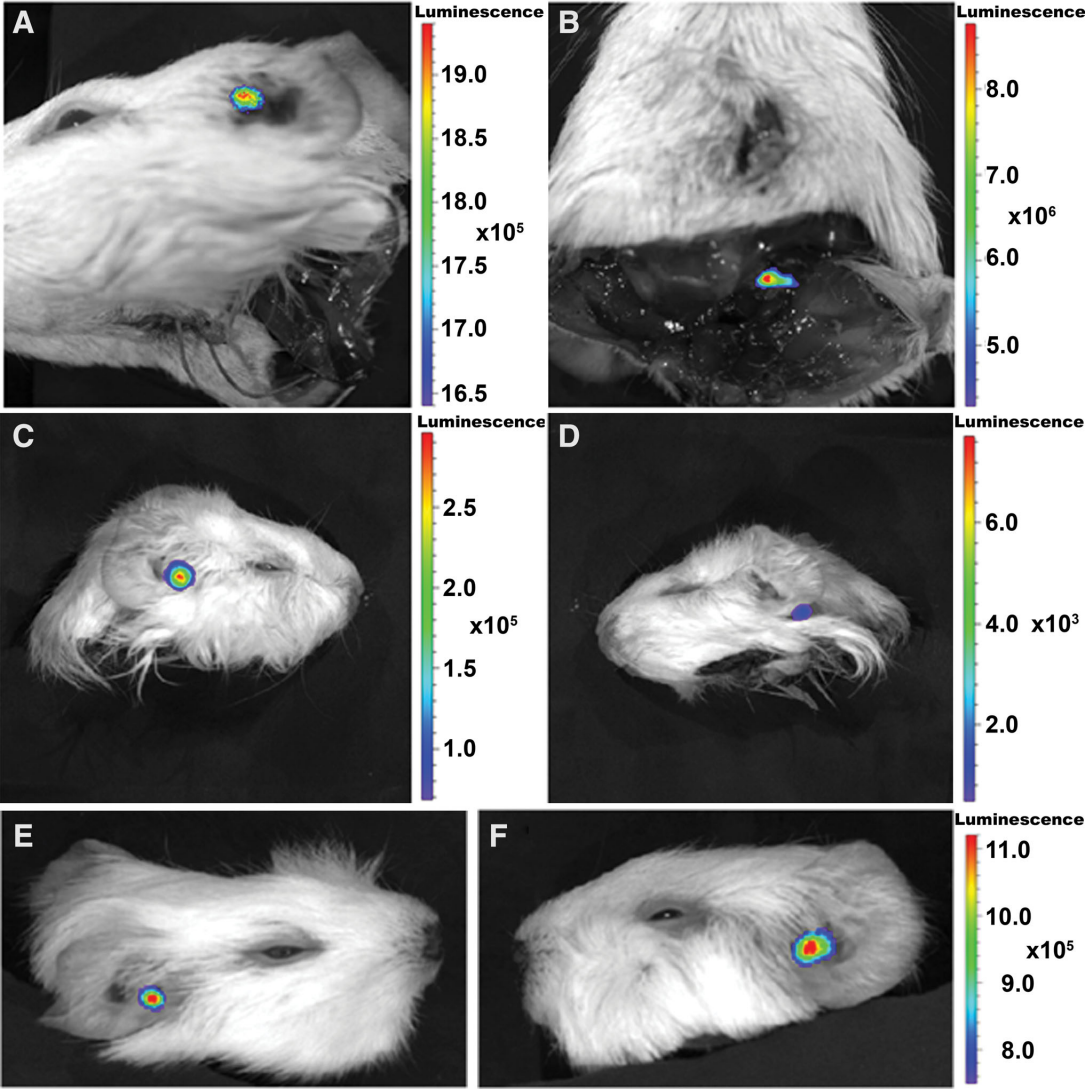
Comparison of the different application routes. (**A**, **B**) Pseudocolor light emission images of guinea pig cadaver heads after injection of HEK293‐copGFP‐CBG99 cells (5 × 10^4^ cells in 10 μL PBS containing D‐luciferin at a final concentration of 15 mg/mL) into the internal auditory meatus. (A) Ventrolateral view of the cadaver head from the left side. Bioluminescent signal is located at the cavum conchae of the auricle. (B) Ventral view of the same head seen from the rear. A distinct bioluminescent signal is seen emanating from the injection site, i.e., the internal auditory meatus. (**C**, **D**) Injection of HEK293‐copGFP‐CBG99 cells (5 × 10^4^ cells in 5 μL PBS containing D‐luciferin) directly into the scala tympani *via* the round window membrane. (C) Lateral view from the right side showing bioluminescent signal located at the cavum conchae of the auricle. (D) Injection of transduced cells into the left masseter muscle results in a considerably lower signal, due to light absorption by tissue chromophores. (**E**, **F**) Injection of HEK293‐copGFP‐CBG99 cells (5 × 10^4^ cells in 5 μL PBS containing D‐luciferin) directly into the modiolus of the basal cochlear turn. After an initial injection in the right ear (E) followed by a second injection in the left ear (F), in both ears a bright bioluminescent signal was invariably located near the cavum conchae of the auricle, indicating that the light emanating from the cochlea is passing through the tympanic membrane and the external auditory meatus. Bioluminescence is expressed as radiance (photons/second/cm^2^/sr).

The auditory bullae were left *in situ* in the cadaver heads and the foramen magnum was widened to allow easy access to the internal auditory meatus. After injection of HEK293‐copGFP‐CBG99 cells into the internal auditory meatus of the right auditory bulla, a bright bioluminescent signal was observed in the cavum conchae of the auricle (Fig. 4A), indicating that light generated by enzymatic (luciferase) conversion of D‐luciferin is passing through the tympanic membrane and the external auditory meatus.

#### *Approach* via *the round window: Direct injection into the scala tympani*

In this experiment, both auditory bullae were left *in situ* in the cadaver heads. After opening the auditory bulla, HEK293‐copGFP‐CBG99 cells were injected through the round window membrane directly into the scala tympani. The bioluminescent signal was invariably located near the cavum conchae of the auricle (Fig. 4C), indicating that the light emitted by the cochlea was passing through the tympanic membrane and the external auditory meatus. Control injection into the masseter muscle resulted in much less bioluminescent signal, due to light absorption by tissue chromophores such as oxyhemoglobin, myoglobin and cytochromes (Fig. 4D).

#### *Approach* via *the round window: Direct injection into Rosenthal's canal*

Similar results were obtained after injection of HEK293‐copGFP‐CBG99 cells through the round window membrane, directly into the modiolus of the basal cochlear turn (Fig. 4E,F). Molecular optical imaging of the auditory bullae following their removal from the skull demonstrated that the bioluminescent signal is passing through the tympanic membrane and crevices in the bony wall of the bullae. After opening the auditory bulla to show its internal structures, a distinct bioluminescent signal was seen emanating from the area near the round window, i.e., the site of injection (data not shown; *cf*., Fig. 3). The finding that the bioluminescent signal was restricted to a small area—rather than being dispersed more widely—indicates that leakage of the fluid after intracochlear injection does not occur.

In future transplantation experiments we aim to introduce transduced stem cells into the spiral ganglion and for that reason injection into Rosenthal's canal is the approach of first choice.

#### In vitro *light‐microscopical detection of reporter molecule expression in HFBSCs with copGFP‐Luc2*

**Figure 5:**
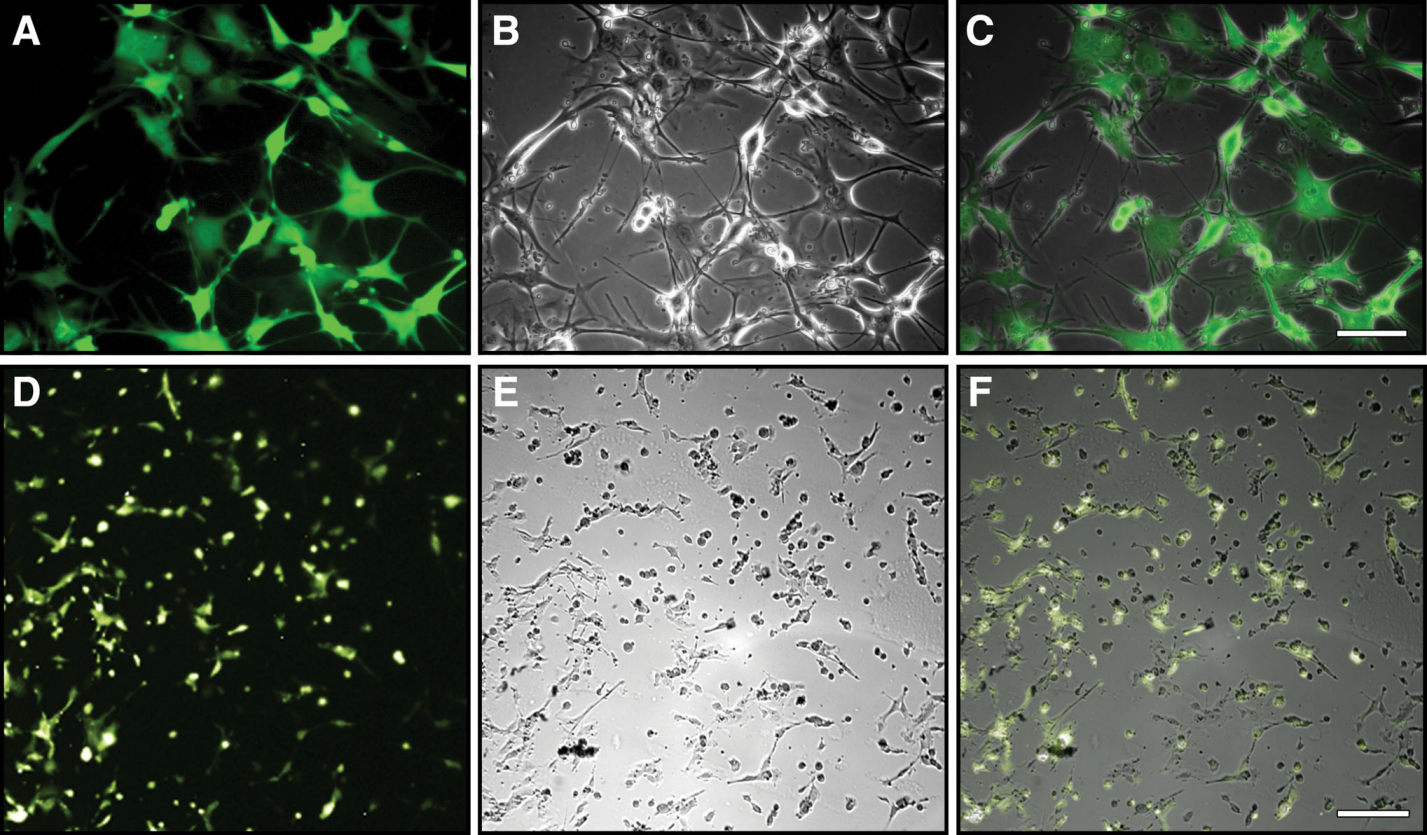
Detection of reporter molecule expression in HFBSCs expressing copGFP‐Luc2. Light micrographs showing reporter molecule expression in cultures of transduced HFBSCs containing the copGFP‐Luc2 construct. (**A**–**C**) Fluorescence microscopy: Fluorescence (A) and bright‐field (B) images of a representative area in the culture were merged and the resulting overlay (C) shows that nearly all cells express the fluorescent reporter molecule copGFP (scale bar: 100 μm). (**D**–**F**) Bioluminescence microscopy: From the overlay (F) of the bioluminescence (D) and bright‐field (E) images it is clear that most (i.e., viable) cells express the bioluminescent reporter molecule Luc2 (scale bar: 200 μm).

Cultures of transduced HFBSCs containing the copGFP‐Luc2 construct were tested for copGFP expression using fluorescence microscopy. Fluorescence micrographs and the corresponding bright‐field overlays demonstrate that nearly all cells express copGFP (Fig. 5A–C).

Luc2 expression was verified by means of bioluminescence microscopy. Close examination of the bioluminescent images and the bright‐field overlays reveals that the majority of cells express the bioluminescent reporter molecule, implying that the transduced cells have retained their viability (Fig. 5D–F).

#### In vitro *imaging of luciferase expression in cultures of HFBSCs with copGFP‐Luc2*

**Figure 6:**
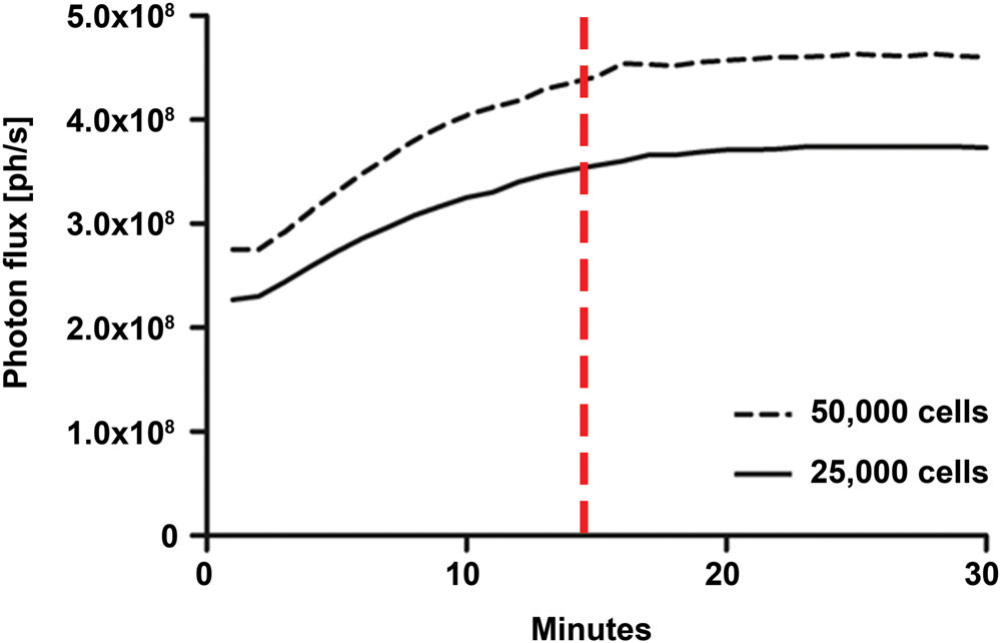
Time course of the bioluminescent signal observed in cultures of transduced HFBSCs. The signal of transduced HFBSCs containing the copGFP‐Luc2 construct gradually increases after addition of D‐luciferin (final concentration: 0.5 mM) and reaches its plateau phase after approximately 15 min (red line), irrespective of the amount of plated cells (dotted line: 5 × 10^4^ cells; solid line: 2.5 × 10^4^ cells) studied. Values for each time point and cell amount represent the averaged data sets from six wells. Bioluminescence is expressed as photon flux (ph/s: photons/second).

Luc2 emission kinetics were measured in cultures of transduced HFBSCs containing the copGFP‐Luc2 construct. Quantitative measurement of Luc2 emission kinetics demonstrates that approximately 15 min after addition of D‐luciferin the bioluminescent signal reaches its plateau phase (Fig. 6), irrespective of the amount of cells (2.5 × 10^4^/100 μL or 5 × 10^4^ cells/100 μL) studied. This image acquisition window was used in all subsequent imaging experiments.

#### *Cell dilution series to determine the limit of detection*

**Figure 7:**
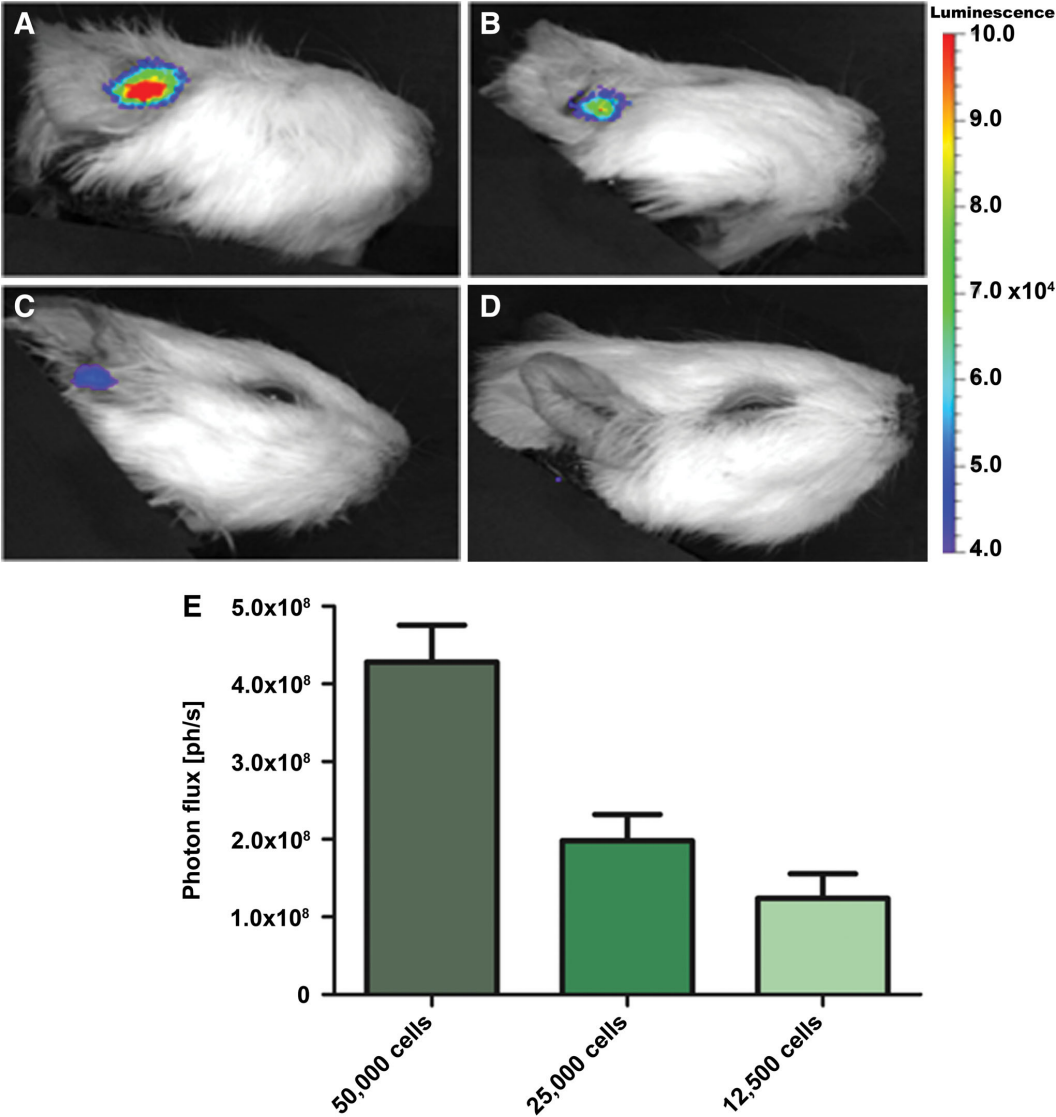
Cell dilution series. (**A**–**D**) To determine the amount of transduced HFBSCs (containing the copGFP‐Luc2 construct) needed to reach signal threshold for bioluminescence imaging a cell dilution series was performed. Bioluminescence is expressed as radiance (photons/second/cm^2^/sr). Cells were resuspended in PBS at a concentration of 1 × 10^6^ cells/100 μL followed by addition of D‐luciferin (final concentration: 15 mg/mL). Different amounts of cells were injected into the modiolus of the basal cochlear turn in the right ear. A bright bioluminescent signal was seen after injection of 5 × 10^4^ cells (A). Considerably lower signals were detected after injection of 2.5 × 10^4^ cells (B) and 1.25 × 10^4^ cells (C). Injection of 0.5 × 10^4^ cells (D) did not result in a detectable bioluminescent signal. (**E**) Quantitative measurement of the bioluminescent signal shows that the threshold for bioluminescence imaging lies between 0.5 × 10^4^ cells and 1.25 × 10^4^ cells. Bioluminescence is expressed as photon flux (ph/s: photons/second).

A cell dilution series was performed to determine the limit of detection for the bioluminescent signal using transduced HFBSCs containing the copGFP‐Luc2 construct. A bioluminescent signal of 4.3 ± 0.5 × 10^5^ photons/second was detected after round window membrane injection of an amount of 5 × 10^4^ cells, while lower signals amounting to 2.0 ± 0.3 × 10^5^ and 1.2 ± 0.3 × 10^5^ photons/second were detected after injection of 2.5 × 10^4^ and 1.25 × 10^4^ cells, respectively (*cf*., Fig. 7A–C). Injection of 0.5 × 10^4^ cells did not result in a detectable bioluminescent signal (Fig. 7D). Quantitative analysis shows that the threshold for bioluminescence imaging lies between 0.5 × 10^4^ cells and 1.25 × 10^4^ cells (Fig. 7E).

#### *Detection of iron‐containing HFBSCs in histological sections of cochleas*

**Figure 8:**
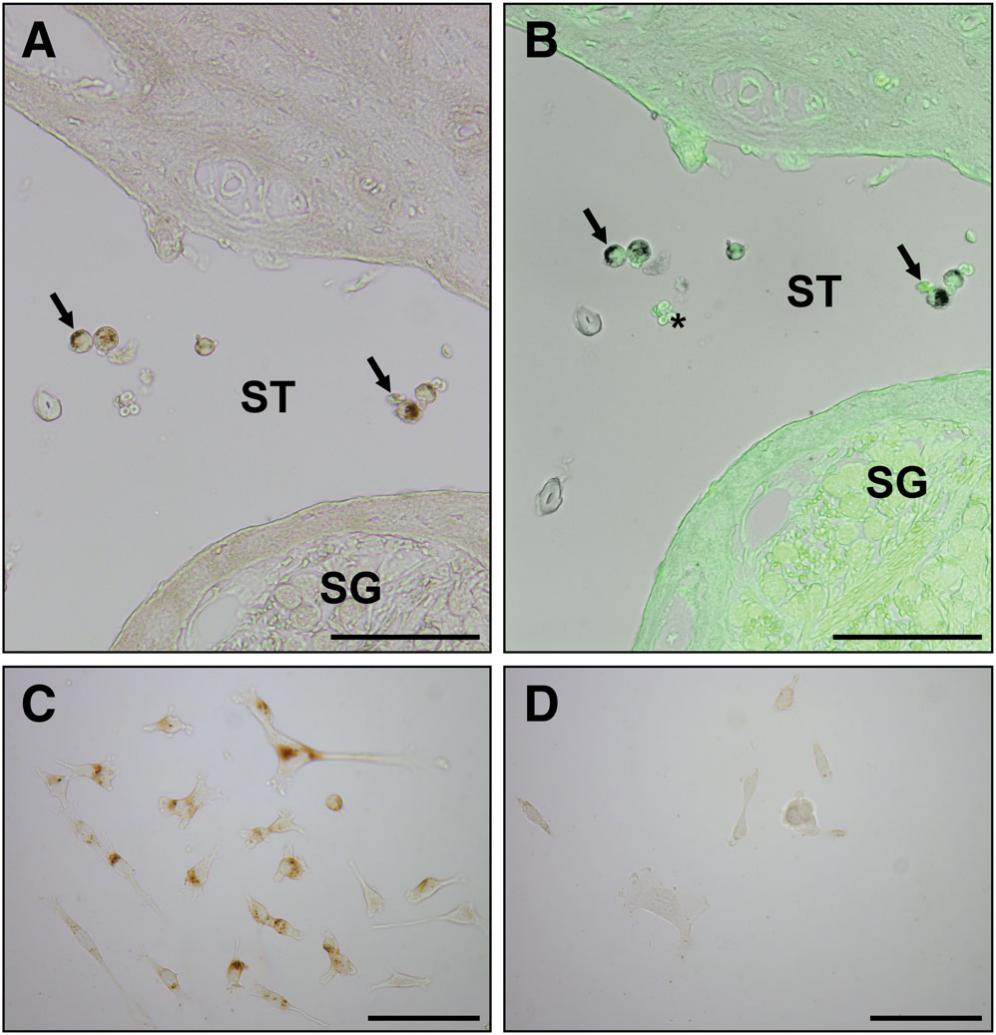
Perls' Prussian blue staining of transduced HFBSCs containing ferumoxytol. Histochemical visualization of non‐heme iron in transduced HFBSCs containing iron oxide nanoparticles (ferumoxytol) using the method with DAB intensification. Dark brown precipitates represent the presence of intracellular iron accumulations. (**A**) Iron accumulations in transduced iron‐containing HFBSCs (arrows) after injection into the scala tympani (ST) of the cochlea. The spiral ganglion (SG) does not contain iron accumulations. (**B**) Same section, but with overlay of grayscale bright field image with the green‐fluorescent signal, demonstrating that iron‐containing HFBSCs also express copGFP (arrows). However, spiral ganglion cells, myelin sheath, bone matrix, and erythrocytes (*) demonstrate autofluorescence at the same wavelengths. (**C)** Iron accumulations in transduced HFBSCs in culture dishes after uploading with ferumoxytol nanoparticles. (**D**) Transduced HFBSCs (control) that have not been uploaded with ferumoxytol nanoparticles do not demonstrate any intracellular deposits after treatment with the Perls' Prussian blue method and DAB intensification (scale bar: 100 μm).

Transduced HFBSCs containing iron oxide nanoparticles could be visualized in dewaxed histological sections of the cochlea using the Perls' Prussian blue method and DAB intensification (Fig. 8A).

CopGFP‐expressing cells in deparaffinized histological sections of the cochlea showed a green‐fluorescent signal. However, most probably due to formaldehyde fixation, several cochlear structures (bony capsule and bony modiolus) and tissues (e.g., spiral ganglion cells and myelin sheaths of the auditory neurons) showed a high cellular autofluorescence in the same range of the spectrum, at which copGFP emits light (Fig. 8B). Therefore, our strategy to load the transduced cells also with iron oxide nanoparticles proved to be prudent. Immunohistochemical detection with specific antibodies to copGFP was therefore not necessary.

## DISCUSSION

The objective of this study was to investigate if visualization of exogenous stem cells within the intact guinea pig cochlea, using molecular optical imaging, is feasible for future *in vivo* stem cell transplantation experiments. To the best of our knowledge, this is the first study that has been undertaken to detect luciferase‐expressing cells in the intact cochlea of guinea pigs using bioluminescence imaging. Although other authors have tracked green‐fluorescent stem cells in histological sections of the cochlea from various rodent species using fluorescence microscopy (Regala et al., 2005; Hu and Ulfendahl, 2006; Sekiya et al., 2006; Bogaerts et al., 2008; Ogita et al., 2009; Kasagi et al., 2013), the present study is the first that uses molecular optical imaging for the non‐destructive *in situ* detection of bioluminescent stem cells injected into an anatomically complex structure such as the cochlea.

Whole‐specimen imaging has been used to make three‐dimensional reconstructions of rodent cochleas using digitized optical sections obtained from a variety of fluorescence‐based microscopical techniques, such as orthogonal‐plane fluorescence optical sectioning microscopy (Voie, 2002; Hofman et al., 2009), confocal laser scanning microscopy (MacDonald and Rubel, 2008; Kopecky et al., 2012; Wrzeszcz et al., 2013), thin‐sheet laser imaging microscopy (Santi, 2011) and, more recently, two‐photon excitation fluorescence microscopy which uses near‐infrared excitation wavelengths (Yuan et al., 2010; Yang et al., 2013). However, none of these approaches can be used in sedated animals as they require *post‐mortem* removal of the cochlea from the auditory bulla and rigorous preparatory steps, involving chemical fixation, decalcification, dehydration and clearing. Non‐destructive or minimally invasive *in vivo* imaging modalities—such as fluorescence intravital microscopy (Shi et al., 2014), fluorescence microendoscopy, optical coherence tomography (Tona et al., 2014), and magnetic resonance imaging—can be used to study cochlear blood flow and anatomy in sedated animals, but do not allow real‐time tracking of cells in living animals. However, recent studies have shown that bioluminescence imaging is a valid and efficient imaging technique, in particular in small rodents (Kanzaki et al., 2012; Meltser et al., 2014; Kanzaki et al., 2015; Park et al., 2016).

Our results demonstrate that light emanating from the cochlea can be detected using bioluminescence imaging. We assume that the light does not penetrate the bony capsule of the cochlea nor the thick bone of the auditory bulla, but emanates from the round window membrane and is then reflected by the inner wall of the bulla and escapes through the tympanic membrane and the external auditory meatus. The spatial resolution of bioluminescence imaging is 3–5 mm with a penetration depth of 1–3 cm (Massoud and Gambhir, 2003; Shah and Weissleder, 2005). The distance from round window to tympanic membrane in the guinea pig measures approximately 2 mm, whereas the distance from the round window to the distal end of the bony part of the external auditory meatus is approximately 5 mm (Voie, 2002). The external auditory meatus, measured from its proximal end at the tympanic membrane to its distal opening, i.e., cavum conchae, including the bony and cartilaginous walls, is approximately 4.45–7.45 mm long, with an average length of 5.77 mm (Wysocki and Sharifi, 2005). These considerations suggest that fluorescence imaging with a spatial resolution of 2–3 mm and a penetration depth of only 5–7 mm (Shah and Weissleder, 2005) cannot be used to visualize cells in the cochlea of guinea pigs. Moreover, it should be emphasized that not only is penetration depth a limiting factor, but also absorbance of light due to tissue chromophores, such as oxyhemoglobin, myoglobin and cytochromes, may result in a reduction of photon yield at or near the site of injection (*cf*., Fig. 4B).

Bioluminescence imaging is a highly sensitive technique, detecting amounts of the reporter molecule as low as 10^−15^–10^−17^ M (Zhao et al., 2010). In contrast, fluorescence imaging detects reporter molecule amounts that are in the range of 10^−9^–10^−12^ M (Zhao et al., 2010). Also in favor of bioluminescence is its signal‐to‐noise ratio which is high for luciferase‐based systems, but low for fluorescent proteins (Choy et al., 2003; Zhao et al., 2010). Furthermore, as a result of codon optimization, the expression of Luc2, is considerably higher than that of the commonly used wild‐type firefly luciferase. *In vivo*, its photon yield is higher than that of the green‐emitting click beetle luciferase CBG99 (Mezzanotte et al., 2013) and its spectrum of emission is more red‐shifted, thus allowing injection of a 10‐fold lower amount of cells, i.e., 10^5^ cells instead of 10^6^ cells.

Temporal resolution of bioluminescence imaging is in the range of minutes, with average image acquisition times of 10 min (Choy et al., 2003). With the settings as used in our study, the bioluminescent signal reaches plateau phase approximately 10–15 min after addition of D‐luciferin to the cell suspension. The plateau phase lasts for approximately 30–40 min. This allows data and image acquisition within a temporal window during which signal emission remains stable. Nevertheless, it should be emphasized that luciferase emission kinetics after injection of the luciferin‐containing cell suspension in the cadaver head will be different from that after intravenous or intraperitoneal injection of D‐luciferin alone in a living animal.

In small animals, such as mice and rats, D‐luciferin at a dose of 150 mg/kg is typically used and this is adequate for bioluminescence imaging of most organs. However, higher concentrations of D‐luciferin resulting in higher signal emission (Aswendt et al., 2013) and new luciferase substrates (e.g., CycLuc1) to maximize photon emission (Evans et al., 2014) have been recently used to improve bioluminescence imaging in specific applications. In the guinea pig, we cannot inject D‐luciferin by means of intravenous injections—because of the absence of a tail (vein)—and shall have to resort to other application routes, such as intraperitoneal injection. In future *in vivo* experiments, longitudinal monitoring of grafted stem cells requires repeated bioluminescence imaging sessions in the same individual and, hence, repeated injections with D‐luciferin. For that purpose, D‐luciferin can be applied either *via* repeated middle ear instillation—as used by Kanzaki et al. (2012) to demonstrate GFAP‐expressing cells in cochleas of transgenic GFAP‐Luc mice—or chronically by means of an osmotic minipump with a large infusion reservoir or by repeated intraperitoneal injections.

The advantage of using the construct coding for Luc2 and copGFP is that both proteins are translated as single proteins in an equimolar ratio as a result of co‐translational cleavage (“ribosome skipping”) mediated by the T2A‐like sequence in the bicistronic construct (Szymczak and Vignali, 2005). Efficiency of transduction can then easily be estimated by using fluorescence microscopy.

We have chosen for copGFP, as it has a higher photon yield as compared to other naturally occurring fluorescent proteins and because of the varied availability of antibodies against this protein, enabling *post‐mortem* immunohistochemical demonstration of injected cells in histological sections.

Alternatively, injected stem cells containing iron oxide nanoparticles (e.g., ferumoxytol) can be detected in histological sections using the Perls' Prussian blue method with DAB intensification. With this method we were able to identify ferumoxytol‐containing HFBSCs after injection in the scala tympani, avoiding autofluorescence of cochlear tissues. However, we did not find iron‐containing HFBSCs within the spiral ganglion in histological sections of the cochleas. Apparently, proper engraftment of cells into the modiolus was not achieved, since we injected the cells in non‐viable tissue of guinea pig cadaver heads. We cannot conclude other than that most cells got flushed out during injection and subsequent tissue fixation and processing steps. In addition, we expect that iron oxide‐containing cells will be easily detectable in living animals by means of magnetic resonance imaging (*cf*. Schomann et al., 2016). However, the aim of this study was to visualize luciferase‐expressing cells within the intact guinea pig cochlea, and in this we succeeded.

In this study, we have used guinea pig cadavers, but we are confident that the transduced cells will also express a high level of bioluminescence in a living animal. This is supported by the *in vitro* finding that transduced HFBSCs express luciferase activity after multiple proliferation cycles over a period of 15 weeks (Schomann et al., 2016), which makes us confident to assume that these cells are capable to retain their ability to express luciferase after cochlear injection in living animals. For implantation purposes, the number of cells that is injected into the cochlear modiolus varies between 5 × 10^4^ and 2 × 10^5^ (Corrales et al., 2006; Ogita et al., 2009; Chen et al., 2012). Our cell dilution experiment shows that the limit of detection for bioluminescence of the transduced HFBSCs is between 0.5 × 10^4^ and 1.25 × 10^4^ cells after injection into Rosenthal's canal in the basal turn of the cochlea. Based upon an initial amount of 2 × 10^5^ cells, this would mean that approximately 5% of the population of grafted cells would suffice for detection. However, one may ask if such a low percentage of surviving cells may ultimately result in a sufficient degree of regeneration, taking into account the massive amount of cell debris and dead cells. This will be studied in a series of stem cell transplantation experiments in living, deafened guinea pigs.

In conclusion, this feasibility study demonstrates that bioluminescence imaging enables visualization of luciferase‐expressing, exogenous stem cells in the intact guinea pig cochlea.

## References

[ar24068-bib-0001] Abbas L , Seppen J , Barry SC , Klinkspoor JH , Katen LJ , Lee SP , Garcia JV , Osborne WRA . 2000 Apical gene transfer into quiescent human and canine polarized intestinal epithelial cells by lentivirus vectors. J Virol 74:7642–7645. 10906219 10.1128/jvi.74.16.7642-7645.2000 PMC112286

[ar24068-bib-0002] Aswendt M , Adamczak J , Couillard‐Despres S , Hoehn M . 2013 Boosting bioluminescence neuroimaging: an optimized protocol for brain studies. PLoS One 8:e55662. 23405190 10.1371/journal.pone.0055662 PMC3566035

[ar24068-bib-0003] Bierer JA , Faulkner KF , Tremblay KL . 2011 Identifying cochlear implant channels with poor electrode‐neuron interfaces: electrically evoked auditory brain stem responses measured with the partial tripolar configuration. Ear Hear 32:436–444. 21178633 10.1097/AUD.0b013e3181ff33ab PMC3082606

[ar24068-bib-0004] Bogaerts S , Douglas S , Corlette T , Pau H , Saunders D , McKay S , Oleskevich S . 2008 Microsurgical access for cell injection into the mammalian cochlea. J Neurosci Methods 168:156–163. 17963843 10.1016/j.jneumeth.2007.09.016

[ar24068-bib-0005] Briaire JJ , Frijns JHM . 2006 The consequences of neural degeneration regarding optimal cochlear implant position in scala tympani: A model approach. Hear Res 214:17–27. 16520009 10.1016/j.heares.2006.01.015

[ar24068-bib-0006] Buijink MR , Almog A , Wit CB , Roethler O , Olde Engberink AHO , Meijer JH , Garlaschelli D , Rohling JHT , Michel S . 2016 Evidence for weakened intercellular coupling in the mammalian circadian clock under long photoperiod. PLoS One 11:e0168954. 28006027 10.1371/journal.pone.0168954 PMC5179103

[ar24068-bib-0007] Chen W , Jongkamonwiwat N , Abbas L , Eshtan SJ , Johnson SL , Kuhn S , Milo M , Thurlow JK , Andrews PW , Marcotti W , et al. 2012 Restoration of auditory evoked responses by human ES‐cell‐derived otic progenitors. Nature 490:278–282. 22972191 10.1038/nature11415 PMC3480718

[ar24068-bib-0008] Choy G , O'Connor S , Diehn FE , Costouros N , Alexander HR , Choyke P , Libutti SK . 2003 Comparison of noninvasive fluorescent and bioluminescent small animal optical imaging. Biotechniques 35(1022–1026):1028–1030. 10.2144/03355rr02 14628676

[ar24068-bib-0009] Coleman B , Hardman J , Coco A , Epp S , De Silva M , Crook J , Shepherd R . 2006 Fate of embryonic stem cells transplanted into the deafened mammalian cochlea. Cell Transplant 15:369–380. 16970279 10.3727/000000006783981819 PMC1810231

[ar24068-bib-0010] Corrales CE , Pan L , Li H , Liberman MC , Heller S , Edge AS . 2006 Engraftment and differentiation of embryonic stem cell‐derived neural progenitor cells in the cochlear nerve trunk: Growth of processes into the organ of Corti. J Neurobiol 66:1489–1500. 17013931 10.1002/neu.20310 PMC2040047

[ar24068-bib-0011] De Almeida PE , Van Rappard JR , Wu JC . 2011 *In vivo* bioluminescence for tracking cell fate and function. Am J Physiol Heart Circ Physiol 301:H663–H671. 21666118 10.1152/ajpheart.00337.2011 PMC3191083

[ar24068-bib-0012] Eppsteiner RW , Shearer AE , Hildebrand MS , DeLuca AP , Ji H , Dunn CC , Black‐Ziegelbein EA , Casavant TL , Braun TA , Scheetz TE , et al. 2012 Prediction of cochlear implant performance by genetic mutation: The spiral ganglion hypothesis. Hear Res 292:51–58. 22975204 10.1016/j.heares.2012.08.007 PMC3461332

[ar24068-bib-0013] Evans MS , Chaurette JP , Adams ST Jr , Reddy GR , Paley MA , Aronin N , Prescher JA , Miller SC . 2014 A synthetic luciferin improves bioluminescence imaging in live mice. Nat Methods 11:393–395. 24509630 10.1038/nmeth.2839 PMC4026177

[ar24068-bib-0014] Gantz BJ , Woodworth GG , Knutson JF , Abbas PJ , Tyler RS . 1993 Multivariate predictors of audiological success with multichannel cochlear implants. Ann Otol Rhinol Laryngol 102:909–916. 8285510 10.1177/000348949310201201

[ar24068-bib-0015] Gho CG , Schomann T , De Groot SC , Frijns JHM , Rivolta MN , Neumann MH , Huisman MA . 2016 Isolation, expansion and neural differentiation of stem cells from human plucked hair: a further step towards autologous nerve recovery. Cytotechnology 68:1849–1858. 26702932 10.1007/s10616-015-9938-x PMC5023559

[ar24068-bib-0016] Gunewardene N , Dottori M , Nayagam BA . 2012 The convergence of cochlear implantation with induced pluripotent stem cell therapy. Stem Cell Rev 8:741–754. 10.1007/s12015-011-9320-0 21956409

[ar24068-bib-0017] Hofman R , Segenhout JM , Wit HP . 2009 Three‐dimensional reconstruction of the Guinea pig inner ear, comparison of OPFOS and light microscopy, applications of 3D reconstruction. J Microsc 233:251–257. 19220691 10.1111/j.1365-2818.2009.03115.x

[ar24068-bib-0018] Hu Z , Ulfendahl M . 2006 Cell replacement therapy in the inner ear. Stem Cells Dev 15:449–459. 16846380 10.1089/scd.2006.15.449

[ar24068-bib-0019] Huisman MA , Rivolta MN . 2012 Neural crest stem cells and their potential application in a therapy for deafness. Front Biosci 4:121–132. 10.2741/s255 22202047

[ar24068-bib-0020] Kalkman RK , Briaire JJ , Frijns JHM . 2015 Current focussing in cochlear implants: An analysis of neural recruitment in a computational model. Hear Res 322:89–98. 25528491 10.1016/j.heares.2014.12.004

[ar24068-bib-0021] Kanzaki S , Fujioka M , Yasuda A , Shibata S , Nakamura M , Okano HJ , Ogawa K , Okano H . 2012 Novel *in vivo* imaging analysis of an inner ear drug delivery system in mice: comparison of inner ear drug concentrations over time after transtympanic and systemic injections. PLoS One 7:e48480. 23251331 10.1371/journal.pone.0048480 PMC3520978

[ar24068-bib-0022] Kanzaki S , Watanabe K , Fujioka M , Shibata S , Nakamura M , Okano HJ , Okano H , Ogawa K . 2015 Novel in vivo imaging analysis of an inner ear drug delivery system: Drug availability in inner ear following different dose of systemic drug injections. Hear Res 330:142–146. 26435094 10.1016/j.heares.2015.09.018

[ar24068-bib-0023] Kasagi H , Kuhara T , Okada H , Sueyoshi N , Kurihara H . 2013 Mesenchymal stem cell transplantation to the mouse cochlea as a treatment for childhood sensorineural hearing loss. Int J Pediatr Otorhinolaryngol 77:936–942. 23561635 10.1016/j.ijporl.2013.03.011

[ar24068-bib-0024] Kopecky BJ , Duncan JS , Elliott KL , Fritzsch B . 2012 Three‐dimensional reconstructions from optical sections of thick mouse inner ears using confocal microscopy. J Microsc 248:292–298. 23140378 10.1111/j.1365-2818.2012.03673.x PMC3625616

[ar24068-bib-0025] Liu F , Uchugonova A , Kimura H , Zhang C , Zhao M , Zhang L , Koenig K , Duong J , Aki R , Saito N , et al. 2011 The bulge area is the major hair follicle source of nestin‐expressing pluripotent stem cells which can repair the spinal cord compared to the dermal papilla. Cell Cycle 10:830–839. 21330787 10.4161/cc.10.5.14969

[ar24068-bib-0026] MacDonald GH , Rubel EW . 2008 Three‐dimensional imaging of the intact mouse cochlea by fluorescent laser scanning confocal microscopy. Hear Res 243:1–10. 18573326 10.1016/j.heares.2008.05.009 PMC2566306

[ar24068-bib-0027] Massoud TF , Gambhir SS . 2003 Molecular imaging in living subjects: seeing fundamental biological processes in a new light. Genes Dev 17:545–580. 12629038 10.1101/gad.1047403

[ar24068-bib-0028] Meguro R , Asano Y , Odagiri S , Li C , Iwatsuki H , Shoumura K . 2007 Nonheme‐iron histochemistry for light and electron microscopy: a historical, theoretical and technical review. Arch Histol Cytol 70:1–19. 17558140 10.1679/aohc.70.1

[ar24068-bib-0029] Meltser I , Cederroth CR , Basinou V , Savelyev S , Lundkvist GS , Canlon B . 2014 TrkB‐mediated protection against circadian sensitivity to noise trauma in the murine cochlea. Curr Biol 24:658–663. 24583017 10.1016/j.cub.2014.01.047 PMC3962718

[ar24068-bib-0030] Mezzanotte L , Aswendt M , Tennstaedt A , Hoeben R , Hoehn M , Löwik C . 2013 Evaluating reporter genes of different luciferases for optimized *in vivo* bioluminescence imaging of transplanted neural stem cells in the brain. Contrast Media Mol Imaging 8:505–513. 24375906 10.1002/cmmi.1549

[ar24068-bib-0031] Mezzanotte L , Van 't Root M , Karatas H , Goun EA , Lowik C . 2017 In vivo molecular bioluminescence imaging: New tools and applications. Trends Biotechnol 35:640–652. 28501458 10.1016/j.tibtech.2017.03.012

[ar24068-bib-0032] Needham K , Minter RL , Shepherd RK , Nayagam BA . 2013 Challenges for stem cells to functionally repair the damaged auditory nerve. Expert Opin Biol Ther 13:85–101. 23094991 10.1517/14712598.2013.728583 PMC3543850

[ar24068-bib-0033] Nguyen TD , Widera D , Greiner J , Muller J , Martin I , Slotta C , Hauser S , Kaltschmidt C , Kaltschmidt B . 2013 Prolonged cultivation of hippocampal neural precursor cells shifts their differentiation potential and selects for aneuploid cells. Biol Chem 394:1623–1636. 24084358 10.1515/hsz-2013-0191

[ar24068-bib-0034] Ogita H , Nakagawa T , Lee KY , Inaoka T , Okano T , Kikkawa YS , Sakamoto T , Ito J . 2009 Surgical invasiveness of cell transplantation into the Guinea pig cochlear modiolus. ORL J Otorhinolaryngol Relat Spec 71:32–39. 18971600 10.1159/000165915

[ar24068-bib-0035] Ogoh K , Akiyoshi R , May Maw T , Sugiyama T , Dosaka S , Hatta‐Ohashi Y , Suzuki H . 2014 Bioluminescence microscopy using a short focal‐length imaging lens. J Microsc 253:191–197. 24386879 10.1111/jmi.12109 PMC4285814

[ar24068-bib-0036] Park JS , Cederroth CR , Basinou V , Meltser I , Lundkvist G , Canlon B . 2016 Identification of a circadian clock in the inferior colliculus and its dysregulation by noise exposure. J Neurosci 36:5509–5519. 27194331 10.1523/JNEUROSCI.3616-15.2016 PMC4871986

[ar24068-bib-0037] Rask‐Andersen H , Bostrom M , Gerdin B , Kinnefors A , Nyberg G , Engstrand T , Miller JM , Lindholm D . 2005 Regeneration of human auditory nerve. *In vitro*/*in video* demonstration of neural progenitor cells in adult human and Guinea pig spiral ganglion. Hear Res 203:180–191. 15855043 10.1016/j.heares.2004.12.005

[ar24068-bib-0038] Regala C , Duan M , Zou J , Salminen M , Olivius P . 2005 Xenografted fetal dorsal root ganglion, embryonic stem cell and adult neural stem cell survival following implantation into the adult vestibulocochlear nerve. Exp Neurol 193:326–333. 15869935 10.1016/j.expneurol.2004.12.027

[ar24068-bib-0039] Santi PA . 2011 Light sheet fluorescence microscopy: a review. J Histochem Cytochem 59:129–138. 21339178 10.1369/0022155410394857 PMC3201139

[ar24068-bib-0040] Schomann T , Mezzanotte L , Lourens I‐A‐LM , de Groot JCMJ , Frijns JHM , Huisman MA . 2016 Lentiviral transduction and subsequent loading with nanoparticles do not affect cell viability and proliferation in hair‐follicle‐bulge‐derived stem cells in vitro. Contrast Media Mol Imaging 11:550–560. 27976505 10.1002/cmmi.1717

[ar24068-bib-0041] Sekiya T , Kojima K , Matsumoto M , Kim TS , Tamura T , Ito J . 2006 Cell transplantation to the auditory nerve and cochlear duct. Exp Neurol 198:12–24. 16376874 10.1016/j.expneurol.2005.11.006

[ar24068-bib-0042] Sekiya T , Kojima K , Matsumoto M , Holley MC , Ito J . 2007 Rebuilding lost hearing using cell transplantation. Neurosurgery 60:417–433. 17327786 10.1227/01.NEU.0000249189.46033.42

[ar24068-bib-0043] Shagin DA , Barsova EV , Yanushevich YG , Fradkov AF , Lukyanov KA , Labas YA , Semenova TN , Ugalde JA , Meyers A , Nunez JM , et al. 2004 GFP‐like proteins as ubiquitous metazoan superfamily: evolution of functional features and structural complexity. Mol Biol Evol 21:841–850. 14963095 10.1093/molbev/msh079

[ar24068-bib-0044] Shah K , Weissleder R . 2005 Molecular optical imaging: applications leading to the development of present day therapeutics. NeuroRx 2:215–225. 15897946 10.1602/neurorx.2.2.215 PMC1064987

[ar24068-bib-0045] Shi F , Edge AS . 2013 Prospects for replacement of auditory neurons by stem cells. Hear Res 297:106–112. 23370457 10.1016/j.heares.2013.01.017 PMC3594553

[ar24068-bib-0046] Shi X , Zhang F , Urdang Z , Dai M , Neng L , Zhang J , Chen S , Ramamoorthy S , Nuttall AL . 2014 Thin and open vessel windows for intra‐vital fluorescence imaging of murine cochlear blood flow. Hear Res 313:38–46. 24780131 10.1016/j.heares.2014.04.006 PMC4176943

[ar24068-bib-0047] Shinomori Y , Dd J , Spack DS , Kimura RS . 2001 Volumetric and dimensional analysis of the Guinea pig inner ear. Ann Otol Rhinol Laryngol 110:91–98. 11201817 10.1177/000348940111000117

[ar24068-bib-0048] Snel‐Bongers J , Briaire JJ , van der Veen EH , Kalkman RK , Frijns JH . 2013 Threshold levels of dual electrode stimulation in cochlear implants. J Assoc Res Otolaryngol 14:781–790. 23695303 10.1007/s10162-013-0395-y PMC3767869

[ar24068-bib-0049] Szymczak AL , Vignali DA . 2005 Development of 2A peptide‐based strategies in the design of multicistronic vectors. Expert Opin Biol Ther 5:627–638. 15934839 10.1517/14712598.5.5.627

[ar24068-bib-0050] Thorne M , Salt AN , DeMott JE , Henson MM , Henson OW Jr , Gewalt SL . 1999 Cochlear fluid space dimensions for six species derived from reconstructions of three‐dimensional magnetic resonance images. Laryngoscope 109:1661–1668. 10522939 10.1097/00005537-199910000-00021

[ar24068-bib-0051] Thu MS , Bryant LH , Coppola T , Jordan EK , Budde MD , Lewis BK , Chaudhry A , Ren J , Varma NR , Arbab AS , et al. 2012 Self‐assembling nanocomplexes by combining ferumoxytol, heparin and protamine for cell tracking by magnetic resonance imaging. Nat Med 18:463–467. 22366951 10.1038/nm.2666 PMC3296876

[ar24068-bib-0052] Tona Y , Sakamoto T , Nakagawa T , Adachi T , Taniguchi M , Torii H , Hamaguchi K , Kitajiri S , Ito J . 2014 *In vivo* imaging of mouse cochlea by optical coherence tomography. Otol Neurotol 35:e84–e89. 24448302 10.1097/MAO.0000000000000252

[ar24068-bib-0053] Voie AH . 2002 Imaging the intact Guinea pig tympanic bulla by orthogonal‐plane fluorescence optical sectioning microscopy. Hear Res 171:119–128. 12204356 10.1016/s0378-5955(02)00493-8

[ar24068-bib-0054] Welsh DK , Noguchi T . 2012 Cellular bioluminescence imaging. Cold Spring Harb Protoc 2012:852–866. 10.1101/pdb.top070607 22854570

[ar24068-bib-0055] WHO . 2017 Deafness and hearing loss. In: WHO Fact Sheet No. 300; http://www.who.int/mediacentre/factsheets/fs300/en/index.html.

[ar24068-bib-0056] Wrzeszcz A , Reuter G , Nolte I , Lenarz T , Scheper V . 2013 Spiral ganglion neuron quantification in the Guinea pig cochlea using Confocal Laser Scanning Microscopy compared to embedding methods. Hear Res 306:145–155. 23968822 10.1016/j.heares.2013.08.002

[ar24068-bib-0057] Wysocki J , Sharifi M . 2005 Measurements of selected parameters of the Guinea pig temporal bone. Folia Morphol 64:145–150. 16228948

[ar24068-bib-0058] Yang X , Pu Y , Hsieh CL , Ong CA , Psaltis D , Stankovic KM . 2013 Two‐photon microscopy of the mouse cochlea *in situ* for cellular diagnosis. J Biomed Opt 18:31104. 23165736 10.1117/1.JBO.18.3.031104

[ar24068-bib-0059] Yuan T , Gao SS , Saggau P , Oghalai JS . 2010 Calcium imaging of inner ear hair cells within the cochlear epithelium of mice using two‐photon microscopy. J Biomed Opt 15:016002. 20210449 10.1117/1.3290799 PMC2821419

[ar24068-bib-0060] Zhao C , Tian M , Zhang H . 2010 *In vivo* stem cell imaging. Open Nucl Med J 2:171–177.

